# Macrophage phenotypes and monocyte subsets after destabilization of the medial meniscus in mice

**DOI:** 10.1002/jor.24958

**Published:** 2020-12-29

**Authors:** Lizette Utomo, Niamh Fahy, Nicole Kops, Sandra T. van Tiel, Jan Waarsing, Jan A. N. Verhaar, Pieter J. M. Leenen, Gerjo J. V. M. van Osch, Yvonne M. Bastiaansen‐Jenniskens

**Affiliations:** ^1^ Department of Orthopaedics, Erasmus MC University Medical Center Rotterdam Rotterdam The Netherlands; ^2^ Department of Oral and Maxillofacial Surgery, Erasmus MC University Medical Center Rotterdam Rotterdam The Netherlands; ^3^ Department of Radiology and Nuclear Medicine, Erasmus MC University Medical Center Rotterdam Rotterdam The Netherlands; ^4^ Department of Immunology, Erasmus MC University Medical Center Rotterdam The Netherlands; ^5^ Department of Otorhinolaryngology, Erasmus MC University Medical Center Rotterdam Rotterdam The Netherlands; ^6^ Present address: L. Utomo, Department of Oral and Maxillofacial Surgery & Special Dental Care, University Medical Center Utrecht, Department of Clinical Sciences, Faculty of Veterinary Medicine Utrecht University Utrecht The Netherlands

**Keywords:** animal model, DMM, macrophages, monocytes, osteoarthritis

## Abstract

Macrophages play an important role in the development and progression of osteoarthritis (OA). The aim of this study was to identify macrophage phenotypes in synovium and monocyte subsets in peripheral blood in C57BL/6 mice by destabilizing the medial meniscus (DMM), and the association of macrophage subsets with OA features. DMM, sham, and non‐operated knees were histologically assessed between 1 and 56 days for macrophage polarization states by immunohistochemistry (IHC), cartilage damage, synovial thickening, and osteophytes (*n* = 9 per timepoint). Naive knees (*n* = 6) were used as controls. Monocyte and polarized synovial macrophage subsets were evaluated by flow cytometry. CD64 and CD206 levels on IHC were higher at early timepoints in DMM and sham knees compared to naive knees. iNOS labeling intensity was higher in DMM and sham knees than in naive knees from d3 onwards. CD163 expression was unaltered at all timepoints. Even though macrophage polarization profiles were similar in DMM and sham knees, only in DMM knees the presence of iNOS and CD206 associated with synovial thickness, and CD163 staining inversely correlated with osteophyte presence. At day 14, monocyte subset distribution was different in peripheral blood of DMM mice compared with sham mice. In conclusion, monocyte subsets in blood and synovial macrophage phenotypes vary after joint surgery. High levels of iNOS^+^, CD163^+^, and CD206^+^ cells are found in both destabilized and sham‐operated knees, and coexistence with joint instability may be a requirement to initiate and exacerbate OA progression.

## 1 INTRODUCTION

Macrophages are present in the synovial lining of joints and are thought to have a prominent role in cartilage degeneration and the development and progression of osteoarthritis (OA).^1^ When tissue macrophages become activated, this can result in a spectrum of phenotypes ranging from pro‐inflammatory (M1‐like), to anti‐inflammatory or tissue repair‐stimulating (M2‐like) macrophages.^2^ M1‐like macrophages have increased microbicidal activity, and secrete inflammatory factors such as interleukin (IL)−1β, IL‐6, and chemokine (C‐X‐C motif) ligand (CXCL)10. M2a‐like tissue‐repair macrophages express and secrete for instance IL‐1RA, CD206, and C‐C motif ligand (CCL)18, and have a role in wound healing, tissue repair, and tissue remodeling. M2c‐like macrophages secrete and express IL‐10 and CD163, and are generally considered immunosuppressive.^3^, ^4^ In several studies, increased levels of pro‐inflammatory cytokines typically secreted by activated macrophages were found in synovial fluid of patients who acquired a traumatic injury and were likely to develop OA.^5^, ^6^ Furthermore, the synovial fluid of patients who already had developed post‐traumatic OA showed elevated cytokine levels that may be ascribed to accumulations of pro‐inflammatory macrophages.^1^, ^7^, ^8^ Accumulation of pro‐inflammatory M1‐like macrophages has been observed in human OA synovial tissue as well as in the collagenase‐induced OA (CIOA) mouse model.^9^ The CIOA model is associated with a higher degree and differential course of synovial inflammation compared with surgical models of OA involving destabilization of the medial meniscus.^10^ In addition, macrophages were shown to be essential for the progression of OA in CIOA mouse models,^11^ as depletion of synovial macrophages was shown to diminish osteophyte formation^12^ and reduce cartilage destruction.^13^

In addition to synovial tissue‐resident macrophages, infiltrating monocytes may also participate in OA pathogenesis. Most tissue‐resident macrophages have an embryological origin and self‐renew when necessary.^14^, ^15^ During steady‐state conditions, circulating monocytes do not contribute to the majority of peripheral tissue macrophage populations. However, under certain inflammatory conditions, monocytes migrate to affected tissues and differentiate to macrophages^16^ and thereby also contribute to pathological processes driving OA progression.^17^ Human monocytes can be divided into three subsets based on cell surface receptor expression: classical (CD14^++^CD16^−^), intermediate (CD14^++^CD16^+^) and non‐classical (CD14^+^CD16^++^) monocytes.^18^ In mice, similar peripheral blood monocyte subsets differing in phenotype and function are distinguished^18^ but are often categorized according to two subsets.^19^ Mouse classical monocytes have high level expression of cell surface proteins Ly6C, CD62L and C‐C motif Chemokine Receptor 2 (CCR2), and are rapidly recruited to the site of infection and inflammation, where they contribute to local inflammatory processes and have proteolytic functions.^20^ The second subset, comprising non‐classical monocytes, shows low level expression of Ly6C and CD62L, and high expression of C‐X3‐C Motif Chemokine Receptor 1 (CX_3_CR1). Non‐classical monocytes exhibit patrolling behavior involving adherence to and migration along vascular endothelium, they may promote angiogenesis and tissue repair processes,^20^ and can extravasate following tissue injury and initiate early inflammatory responses.^21^ It was previously reported that classical monocytes were mobilized to synovium 7 days after induction of OA by collagenase injection into mouse knees.^22^ Although perturbation of peripheral blood monocytes subsets has been observed in association with various inflammatory conditions,^23^ their responsiveness to OA development requires further elucidation.

Thus, knowledge of macrophage and monocyte subset kinetics in vivo could provide insights important for therapy. Therefore, the aims of this study were to determine how macrophage phenotypes and monocyte subsets vary with time after destabilization of the medial meniscus and to investigate associations between macrophage phenotypes and OA features.

## 2 METHODS

### 2.1 Induction of experimental OA in mice by DMM

The animal experiments were carried out with the approval of the Animal Ethical Committee of the Erasmus University Medical Center, approval no. EMC 3246 (114‐14‐01) and in accordance with the ARRIVE Guidelines for reporting animal research.^24^ Male C57BL/6 mice (C57BL/6J01aHsd, 12–14 weeks old, 28.4 ± 3.1 g; Envigo, Cambridgeshire, UK) were randomly taken from their cages for induction of OA by destabilizing the medial meniscus (DMM). Mice were anesthetized using 3% isoflurane/0.8 L O_2_/min (Pharmachemie) and received subcutaneous 0.05 mg/kg Temgesic (RB Pharmaceuticals) analgesic 30 min before the procedures. The medial meniscotibial ligament (MMTL) was transected as described by Glasson *et al*.^25^ The contralateral knees underwent a sham procedure which entailed the same procedure, with the exception of transection of the MMTL. To take into account differences in biomechanics and gait, six naive knees were obtained from three 16‐week‐old mice that were euthanized without OA induction. Since there were no previous findings reported regarding the standard deviation of the presence of macrophages during OA, and the use of ranks as nonparametric readout parameter, no sample size calculation was conducted. About 9–12 animals per group were used here as this is a common average sample size used in OA studies.^26^, ^27^ All animals were housed in groups of 3–9 in individually ventilated cages including enrichment under a standard 12 h light/dark cycle at the Experimental Animal Facility of the Erasmus University Medical Center. The animals received acid tap water and standard chow ad libitum. The mice were euthanized by cervical dislocation 1, 3, 7, 14, 28, or 56 days after induction of OA and the knees were processed for histological analysis. As a number of knees were lost during processing (i.e., during harvesting, sectioning, or staining), the datapoints of the individual knees, representing the number of knees used for analysis, are shown in each figure separately.

### 2.2 Histological analysis

The knees were fixed in a 120° flexed position and processed for histological analysis, as described in additional detail in the supplementary methods. The knees were embedded in paraffin and sectioned serially at 6 µm. For each staining a minimum of six sections per knee with less than 180 µm intervals from anterior to posterior were collected on hydrophilic glass slides (StarFrost, Knittel Glass). Sections from all knees were stained with 0.04% thionin in 0.01 M aqueous sodium acetate, pH 4.5 (Sigma‐Aldrich) to evaluate glycosaminoglycans in cartilage, structural cartilage damage, synovial thickness, and the presence of osteophytes. Sections of all knees were immunohistochemically stained in one batch to visualize CD64, inducible nitric oxide synthase (iNOS), CD163, and mannose receptor C type 1/CD206 to identify different macrophage phenotypes.

### 2.3 Scoring of histopathological features of OA and macrophage phenotypes

Structural cartilage damage was assessed in four quadrants (i.e., medial femoral condyle, medial tibial plateau, lateral femoral condyle, and lateral tibial plateau) of four thionin‐stained sections, and evaluated according to a modified Pritzker score^28^ to be more suitable for scoring articular cartilage damage in mouse knees, as well as with the OARSI score^29^ (Table S1). The Pritzker score was determined by multiplying a grade (0–6) and a stage (0–4; Figure S1). Both scoring methods were applied using four sections per quadrant, accounting for a total of 16 scores throughout the entire knee joint. The maximum score of each quadrant was summed to determine the total maximum damage in the knee with a maximal possible score of 96 for the Pritzker score and 24 for the OARSI score. Osteophytes were assessed on the medial and lateral side of the knees and were described as either cartilaginous or bony. Synovial thickness was measured medially and laterally at the height of the parapatellar recess at three locations in four sections using NDP.view v2.6.8 (Hamamatsu), accounting for a total of 24 measurements per knee. The mean of all measurements was used to determine the average synovial thickness of the entire knee.

Of the sections immunohistochemically stained for presence of CD64, iNOS, CD163, and CD206, six to seven sections per knee of the same anatomical regions were ranked based on the increasing intensity in the entire knee. Meaning that all knees including contralateral knees and naive knees, were ranked from least intensely to most intensely stained evaluated by bright field microscopy. Knees that exhibited the same amount of positivity were assigned the median rank of the number of equally ranked knees. This resulted in a maximum score of 111 for CD64, iNOS, and CD163, and 112 for CD206. These numbers varied due sample loss during harvesting, sectioning, or staining. All scorings and rankings were performed in a blinded manner by two independent researchers.

### 2.4 Flow cytometric analysis of peripheral blood monocytes and synovial tissue

To obtain longitudinal data regarding the monocyte subsets in peripheral blood, OA was induced by DMM in a second set of mice and the sham procedure was performed in a third set of mice (11–12 mice per group). Peripheral blood was obtained from the facial vein 1, 7, 14, 28, and 56 days after joint surgery. Blood taken 7 days before surgery was considered as baseline. About 50 µl of whole blood was pre‐incubated with purified rat antimouse CD16/CD32 (Mouse BD Fc Block; BD Biosciences) for 5 min on ice, and stained for cell surface expression of CD45, CD11b, CD115, CD62L, and Ly6C (All from BioLegend; Table S2) to identify myeloid cells and specific monocyte subsets. Cells were stained with antibodies for 15 min on ice in the dark, and erythrocytes were lysed following incubation with 2 ml of 1× FACS lysing solution (BD Biosciences) for 10 min. Following centrifugation at 400 *g* for 10 min, supernatant was removed and the resulting cell pellet was washed twice and resuspended in FACSFlow buffer (BD Biosciences). For flow cytometric analysis of synovial macrophages, the patella with surrounding synovial tissue was removed from each knee joint and subjected to enzymatic digestion (8 mice per group). Tissue was incubated with 2.4 mg/ml Dispase II (Roche), 2 mg/ml collagenase Type IV (Life Technologies) and 0.2 mg/ml DNase I (Sigma‐Aldrich) in Hanksʼ Buffered Salt Solution (Thermo Fisher Scientific) at 37°C for 1 h. Following digestion, the cell suspension was filtered through a 100 µm cell strainer. Cells were spun at 400 *g* for 5 min, supernatant was removed and the resulting cell pellet washed and resuspended in FACSFlow buffer. Cells were stained according to Table S2 for cell surface expression of CD11b, F4/80, CD86, and CD206 (All BioLegend) to identify synovial macrophages, and a LIVE/DEAD™ Fixable Dead Cell Stain (1:1000 dilution; Life Technologies) for exclusion of dead cells. Cells were stained for 30 min on ice in the dark, washed with FACSFlow and spun at 400 *g* for 5 min. The supernatant was removed and the resulting cell pellet was washed and resuspended in FACSFlow. Samples were analyzed using a FACSJazz cytometer (BD Biosciences), with 20,000 events recorded per sample. Cellular viability of all digested synovial tissue samples ranged from 69.16 ± 7.59%. Date were analyzed using FlowJo software version 10.0.7 (FlowJo LLC). The applied gating strategies for synovial macrophages and blood monocytes are presented in Figures S2 and S3.

### 2.5 Statistics

Calculations for rank analysis were conducted with MS Excel 2016 (Microsoft) and statistical evaluation was conducted with IBM SPSS 23.0 (IBM). Differences in cartilage damage and synovial thickness between naïve knees and DMM‐knees at each timepoint and sham and DMM‐operated knees were evaluated by Kruskall–Wallis tests followed by Bonferroni post hoc tests. Differences in intensity of the macrophage markers CD64, iNOS, CD163, and CD206 were evaluated between naïve knees and DMM‐operated knees by Kruskall–Wallis tests followed by Bonferroni post hoc.

The presence of osteophytes was statistically assessed with Fisherʼs exact test followed by Bonferroni correction. Differences in blood monocyte subsets between DMM and sham mice were evaluated per time‐point by Mann–Whitney‐U tests.

Correlation analysis was conducted to evaluate associations between the Pritzker and OARSI scores for cartilage damage. Also associations between intensities of macrophage markers (i.e., iNOS, CD163, and CD206) in DMM‐knees or percentages of peripheral blood monocyte subsets and features of OA were evaluated. For the correlations, nonparametric Spearmanʼs rho correlation tests were conducted including bootstrap‐based calculations for the 95% confidence interval (95% CI). Bonferroni correction was applied where needed. Correlation strengths based on the correlation coefficients (*ρ*_s_) were defined as follows: 0.0–0.19: very weak; 0.20–0.39: weak; 0.40–0.59: moderate; 0.60–0.79: strong; 0.80–1.00: very strong.

Discrete data are presented as median, whereas continuous data are presented as the mean. The Bonferroni‐corrected *p*‐values are denoted in the figures and tables, and differences are considered statistically significant for *p* < 0.05.

## 3 RESULTS

### 3.1 OA development over time in the DMM model

Structural cartilage damage was evaluated with both the Pritzker^28^ and OARSI^29^ scoring method, and appeared to be generally mild over time (Figure S4). scoring methods had a significant, strong correlation with each other (*ρ*_s_ = 0.639) for the total cartilage damage within the joint (Figure S5). Since the Pritzker score is more sensitive to mild structural changes, this score was used for further analysis.

**Figure 1:**
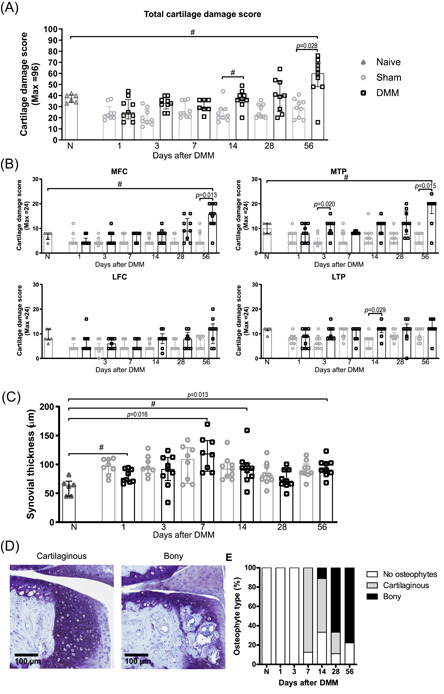
Development of OA features in the DMM mouse model. (**A**) Total structural cartilage damage as scored with the Pritzker method within the entire joint over time and (**B**) per knee compartment. Each symbol represents an individual knee and data is shown as median ± IQR. ^#^: *p* = 0.05–0.10 (**C**) The average synovial thickness as measured at the patella femoral recess. The data are shown as mean ± SD. ^#^: *p* = 0.05‐0.10. (**D**) Osteophytes were identified as either cartilaginous or bony in the thionin‐stained sections. (**E**) Distribution of the osteophyte types in the naive and DMM knees. Each symbol represents an individual knee. LFC: lateral femoral condyle; LTP: lateral tibial plateau; MFC: medial femoral condyle; MTP: medial tibial plateau [Color figure can be viewed at wileyonlinelibrary.com]

After DMM surgery, total cartilage damage at end‐point was significantly more severe in the knees with DMM than in sham‐operated knees (Figure 1A). The difference between naïve joints at 16 weeks of age and DMM‐operated knees was evident, though it did not reach significance (*p* = 0.08). In addition, cartilage damage was significantly increased in the MTP and the MFC of DMM‐knees compared with sham‐operated at day 56 postsurgery (Figure 1B). The synovium was significantly thicker 7 and 56 days after induction of DMM compared with naive knees, but not different from sham‐operated knees, as expected (Figure 1C). No cartilaginous or bony osteophytes (Figure 1D) were present in naive knees, but appeared from day 7 onwards in the majority of the DMM knees. Of the knees in which osteophytes appeared, all were cartilaginous on day 7 and the percentage of bony osteophytes increased from day 28 on (Figure 1E). One out of nine sham‐operated knees had bony osteophytes at day 28, and two out of nine sham‐operated knees had cartilaginous osteophytes at Day 56 (data not shown).

### 3.2 Profiles of macrophage phenotypes after DMM surgery and their correlation with OA features

**Figure 2:**
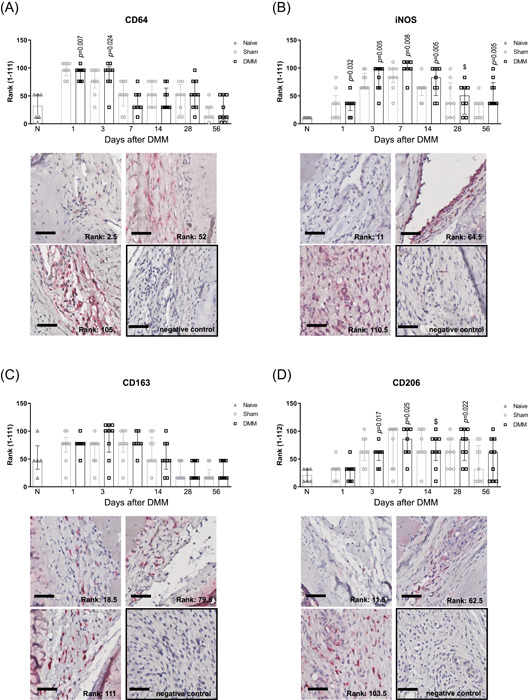
Macrophage phenotypes in the synovial membrane after DMM in mouse knee joints. (**A**) CD64 was used as a pan‐macrophage marker, (**B**) iNOS was used as a pro‐inflammatory (M1‐like) macrophage marker, (**C**) CD163 as a marker for resident and anti‐inflammatory (M2c‐like) macrophages, and (**D**) CD206 as a tissue repair (M2a‐like) macrophage marker. The staining intensity of the markers was ranked amongst all timepoints and statistically compared with the median rank of naive knees that had no surgery (bars labeled with N). The data are shown as median ± IQR and the histological pictures depict examples of three rank levels (DMM or sham knees) including the negative isotype (IgG) control for each marker. Scale bar: 50 µm. The Bonferroni‐adjusted *p*‐values depict statistically significant differences between DMM knees compared to naive knees. ^$^: *p* = 0.05‐0.10. [Color figure can be viewed at wileyonlinelibrary.com]

The intensity of CD64 staining, a pan‐macrophage marker, was higher in DMM than in naive knees (Figure 2A) during the first three days. in DMM knees, the intensity of iNOS staining in the synovial membrane, indicative of of M1‐like macrophage polarization, was significantly higher than in naive knees during almost the entire experiment (Figure 2B). CD163, expressed by several resident macrophage populations and upregulated upon M2c‐like macrophage activation, was present throughout the experiment, but the intensities did not differ significantly from naive knees (Figure 2C). CD206 staining, related to M2a‐like activation, was statistically higher in DMM knees than naive knees at days 3, 7, and 28 (Figure 2D). Sham‐operated knees, however, demonstrated clear staining as well and no significant differences in staining intensity of any of the markers were found between DMM knees and sham‐operated knees. Isotype controls were negative for all stainings (Figure 2).

Associations between IHC staining intensity and OA features were evaluated. iNOS staining in the synovial membrane of DMM knees had a strong positive correlation with synovial thickness (Table 1). CD163 staining had a moderate inverse correlation with the presence of osteophytes and CD206 staining correlated weakly with synovial thickness. Synovial thickening did not correlate with cartilage damage nor with the presence of osteophytes in this model (data not shown). In sham knees, iNOS, CD206, and CD163 stainings did not correlate with any of the OA features, even though similar staining intensities were seen in sham‐operated knees and in DMM knees (Figure 2). iNOS and CD206 staining correlated moderately positive with each other (in DMM knees: *ρ*_s_ = 0.51; 95% CI [0.253–0.709]; in sham knees: *ρ*_s_ = 0.49; 95% CI [0.239–0.699]).

**Table 1:** Spearman rho correlations between staining intensity of macrophage phenotypes in the synovium of DMM knees and OA features

**Macrophage marker**	**Parameter**	**Correlation coefficient (Spearman rho)**	***p*‐value**	**95% CI (lower, upper)**
iNOS (*n* = 51)	Cartilage damage	−0.13	1.000	−0.387, 0.164
	Presence of osteophytes	0.13	1.000	−0.118, 0.374
	**Synovial thickness**	**0.62**	**<0.001**	**0.423, 0.770**
CD163 (*n* = 52)	Cartilage damage	−0.31	0.071	−0.552, −0.017
	**Presence of osteophytes**	**−0.45**	**0.002**	**−0.664, −0.203**
	Synovial thickness	0.19	0.510	−0.088, 0.440
CD206 (*n* = 52)	Cartilage damage	−0.09	1.000	−0.387, 0.228
	Presence of osteophytes	0.28	0.141	−0.018, 0.573
	**Synovial thickness**	**0.35**	**0.035**	**0.053, 0.606**

*Note*: Parameters in bold denote a statistically significant correlation. In the sham knees, the iNOS and CD163 stainings did not correlate with any of the OA features, and CD206 staining had a weak inverse correlation with mild structural cartilage changes.

**Figure 3:**
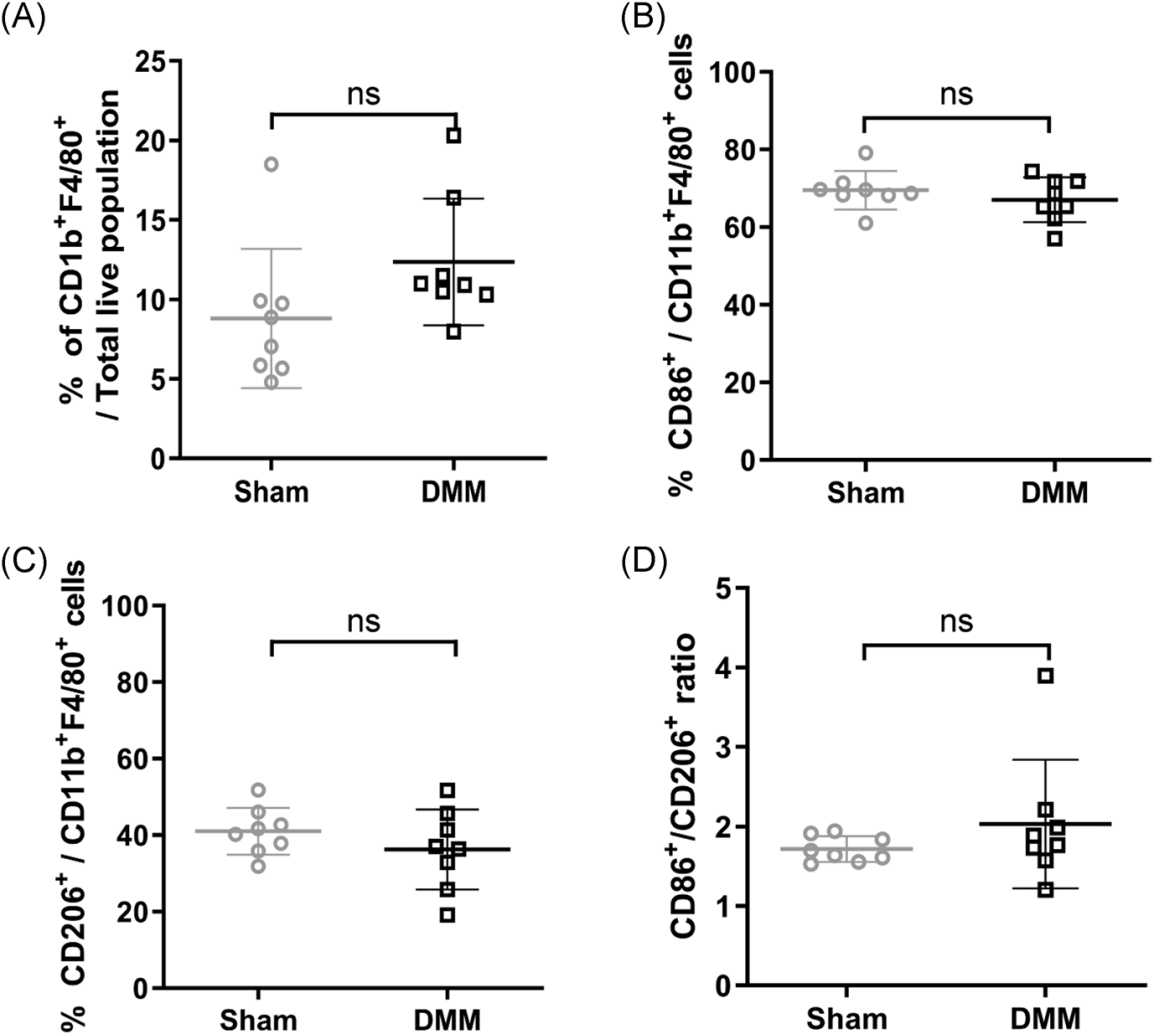
Pro‐inflammatory and tissue repair macrophages in the synovial membrane of knees of mice 56 days after sham and DMM surgery. (**A**) The content of CD11b^+^/F4/80^+^ macrophages as fraction of total synovial cells, (**B**) the percentage of pro‐inflammatory macrophages (CD11b^+^F4/80^+^CD86^+^), (**C**) tissue repair macrophages (CD11b^+^F4/80^+^CD206^+^), and (**D**) the ratio between the percentage of pro‐inflammatory macrophages and tissue repair macrophages in the synovial membrane of sham‐operated mice (*n* = 8) and DMM mice (*n* = 8) 56 days after induction. Each dot represents data of an individual mouse and includes the mean ± SD. *Ns*, not significant

Lastly, the overall content of synovial macrophages and their phenotype was evaluated by flow cytometry at the experimental endpoint. In line with CD64 staining intensity (Figure 2A), similar percentages of CD11b^+^F4/80^+^ synovial macrophages were observed in knees of DMM mice and knees of sham‐operated mice (Figure 3A). Furthermore, in accordance with iNOS and CD206 immunohistochemical staining, the percentages of pro‐inflammatory (CD86^+^/CD11b^+^F4/80^+^) and tissue repair (CD206^+^/CD11b^+^F4/80^+^) macrophages as well as the ratio between both phenotypes, did not significantly differ between DMM and sham‐operated knees (Figure 3B‐D).

### 3.3 Peripheral blood monocyte subsets and synovial macrophages after joint surgery

**Figure 4:**
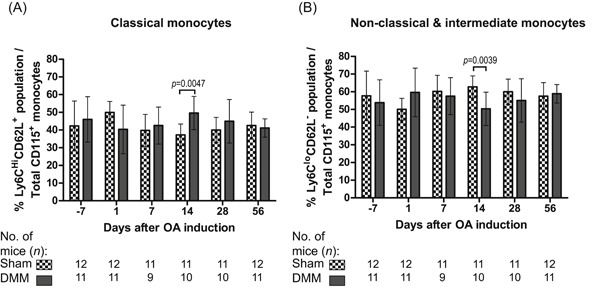
Monocyte subsets in the peripheral blood after DMM and sham surgery. Longitudinal data of the percentage of (**A**) classical monocytes (CD115^+^Ly6C^hi^CD62L^+^) and (**B**) non‐classical and intermediate monocytes (CD115^+^Ly6C^lo^CD62L^−^) subsets. The subsets were identified 7 days before surgery (day −7; baseline) and then in time till 8 weeks after surgery. The data are presented as the mean ± SD and the *p*‐values denote significant differences between DMM and sham knees

To evaluate whether local joint inflammation would be reflected in systemic alterations in circulating monocytes, the percentages of monocyte subsets in peripheral blood were analyzed longitudinally after joint surgery by flow cytometry in a separate experiment. In general, minimal shifts were observed between monocyte subset distributions during the course of the experiment. Yet, percentages of CD115^+^Ly6C^hi^CD62L^+^ classical monocytes (Figure 4A) were statistically significantly higher at day 14 in peripheral blood of DMM mice than in sham‐operated mice, consequently resulting in lower percentages of CD115^+^Ly6C^lo^CD62L^−^ non‐classical monocytes (Figure 4B).

## 4 DISCUSSION

Macrophages have been acknowledged for their role in OA development.^12^, ^13^, ^30^ Profiles of the macrophage phenotypes appeared very similar in DMM knees and in sham knees, but were different from naïve knees. This implies that macrophages are activated due to the injury of tissue such as synovium and the infrapatellar fat pad as result of the surgical procedure and this activation appears not to be linked specifically to the destabilized medial meniscus. Since sham operated knees did not develop features of OA and correlations with macrophages markers were absent in the sham joints, these finding indicates that increased presence of macrophages on its own does not necessarily lead to OA. Biomechanics are known to play a role in promoting inflammation and progression of OA,^31^ and the altered joint mechaniscs in DMM knees is a requirement to initiate the disease.

Previously, it was shown that iNOS^+^ M1‐like macrophages are involved in pro‐inflammatory processes,^32^, ^33^ CD206^+^ M2a‐like macrophages have high production of factors involved in tissue repair processes^34^, ^35^ and CD163^+^ M2c‐like macrophages produce high levels of IL‐10 and soluble CD163 and are typically considered anti‐inflammatory.^36^ We evaluated the overall presence of these three different markers, which are indicative for macrophages with different phenotypes, over time after DMM and sham surgery in mice, and their association with key OA features, synovial thickness, cartilage damaged and osteophytes. Even though the presence of CD163^+^ cells was not different between sham and DMM, the presence of CD163^+^ cells did inversely correlate with the presence of osteophytes within individual mice that underwent DMM. These findings are in line with our previous study, where an increase in CD163‐expressing macrophages induced by intra‐articular injection of the corticosteroid triamcinolone acetonide was linked with reduced osteophyte formation in an OA rat model induced by papain injections combined with a running protocol.^37^ CD206‐expressing cells were continuously present in relatively high amounts following joint surgery. This predominance suggests that CD206^+^ cells were likely compensating and repairing the injury caused. This is supported by the positive association found between CD206^+^ macrophages, known to produce fibrosis‐associated CCL18,^4^, ^34^, ^35^ and synovial thickening in DMM joints. Having identified these profiles provides valuable indications regarding the interplay between joint tissues and macrophages, and the role of inflammation involving monocytes and macrophages during DMM‐induced OA.

In the present study, the intensity of CD64 staining was used as a pan macrophage marker,^38^, ^39^ which highlighted an increase in the level of macrophages in the synovium of DMM‐operated knees compared to naïve knees within the first 3 days postsurgery. Furthermore, staining intensity did not significantly differ between sham and DMM‐operated knees, suggesting that macrophage levels may increase in the synovium of both DMM and sham‐operated knees as an early response to joint injury and subsequently resolve. Single iNOS, CD206, and CD163 staining were assessed to determine potential changes in synovial macrophage polarization state during OA development. However, these markers may also be expressed by other cell types including the expression of CD206 by CD11b^+^ dendritic cells^38^ and iNOS and CD163 expression by monocytes,^40^, ^41^ and the use of single marker staining is a limitation of our study. Further investigation may be required to fully confirm that the composition of macrophage phenotypes is altered in response to the development of OA features over time. Immunohistochemistry comes with additional limitations as the staining intensity does not account for the number of cells present in the tissue. However, the data generated by flow cytometric analysis provides some additional information to the immunohistochemical stainings. Confirming the immunohistochemical analyses of the time course study, we found no differences in M1‐like F4/80^+^/CD86^+^‐macrophages and M2a‐like F4/80^+^/CD206^+^‐macrophages between DMM‐ and sham‐operated mice at day 56. The CD86^+^/CD206^+^‐ratio of synovial macrophages showed a trend towards more CD86^+^ pro‐inflammatory than anti‐inflammatory macrophages in the synovium of DMM and sham knees. However, there was no significant difference in this ratio between DMM and sham‐operated mice where features of OA did not develop, suggesting that it may be a result of surgical intervention, rather than OA feature development resulting from destabilization of the medial meniscus.

We further investigated whether local joint changes would be reflected in changes in circulating monocyte subset levels. Perturbation of peripheral blood monocyte subsets has been previously reported in association with various inflammatory conditions including asthma, aseptic implant loosening, rheumatoid arthritis, and coronary artery disease.^23^ Generally, however, frequencies of classical and non‐classical/intermediate monocytes in peripheral blood were not significantly different between DMM‐ and sham‐treated mice for most timepoints. Only at 14 days after joint surgery, the percentage of classical monocytes in blood was higher with the percentage of non‐classical/intermediate monocytes consequently lower in DMM‐‐operated mice. This may indicate that the local joint inflammation influences circulating monocyte subsets, as it has been shown that non‐classical monocytes initiate joint inflammation in a rheumatoid arthritis model.^42^ Also, classical monocyte recruitment mediated by CCL2/CCR2 signaling has been shown to further propagate tissue damage and inflammation in the DMM model.^17^ A limitation of this study is, however, the high variability in monocyte subset distribution in the mice over time. A high variability in monocyte subset frequencies among wild‐type C57BL/6 mice was observed previously and that report also highlighted an impact of sham surgeries on monocyte subset kinetics in other experimental models.^43^ In light of these findings, a longitudinal comparison to a naive control group or the use of an OA model which minimizes surgery‐induced tissue injury in the sham control group, would be required to elucidate the relationship of monocyte subsets to OA progression more clearly.

The main goal of this study was to evaluate the presence of monocyte and macrophage phenotypes after destabilization of the medial meniscus and examine whether macrophage phenotypes correlate with joint pathology over time. As differences in gait may contribute to joint degeneration in contralateral knees,^44^ which were sham operated, we compared the macrophage phenotype profiles to the profiles of naive knees in which no OA was induced. A limitation of our study is that the naive knees represent only one time point, and it should be taken into account that the naive mice were 16 weeks of age (the same age as the 28 day OA mice), a timepoint where the first changes due to ageing can be expected.^45^ Cartilage damage resulting from DMM surgery became apparent after 56 days compared with sham‐operated knees, and was mainly observed in the MTP and MFC. However, chondrophyte formation was clear from day 7 onwards in the majority of DMM‐operated knees and from day 28 osteophytes were present in the majority. This confirms OA was developing, though slowly. The slow development of OA might be seen as one of the advantages of this model, since it makes it more similar to human OA. In a similar manner to our findings, previous studies have highlighted the slowly progressive nature of the DMM model in C56BL/6 mice, with mild cartilage damage evident by 8 weeks post‐DMM surgery.^46^ In addition, other groups have reported that structural cartilage damage was not significantly increased in knees of C57BL/6 mice following DMM surgery compared with sham‐operated mice after 2 and 4 weeks.^47^, ^48^ In line with our observations, Loeser *et al*.^47^ also found that osteophyte formation was the earliest structural change observed in DMM‐operated joints. The aforementioned studies have implemented different articular cartilage scoring methodologies including the Articular Cartilage Structure score^47^ and OARSI histopathology guidelines.^46^, ^48^, ^49^ In our study, we have chosen to use the Pritzker cartilage damage scoring method as we find it more sensitive for the evaluation of structural cartilage damage since it takes into account both a stage and a grade. Moreover, this scoring method has been used over the years in various other studies, also for the DMM model in C57BL/6 mice^10^, ^27^, ^50^ and our findings regarding the rate of progression of cartilage damage are in line with these previous results, demonstrating that the outcome is consistent with other scoring methodologies.

Although pronounced cartilage damage was not observed in DMM‐operated joints, only in DMM knees, not in the sham‐operated knees, macrophage phenotypes are correlated with specific pathological features of OA: iNOS and CD206 expression associaties with synovial thickness and CD163 expression inversely correlates with the presence of osteophytes. These findings indicate that macrophages alone are unlikely to play a role in the induction of OA features in this model, but might contribute to progression when OA is induced by another stimulus, such as mechanical instability of the joint. Further in vivo investigation should be conducted to assess whether suppressing the activity of pro‐inflammatory macrophages or stimulating anti‐inflammatory macrophages, perhaps via modulation of monocyte subsets, will inhibit OA progression.

## CONFLICT OF INTERESTS

All the authors declare that there are no conflict of interests.

## ETHICAL APPROVAL

The animal experiments were carried out with the approval of the Animal Ethical Committee of the Erasmus Medical Center, Rotterdam, the Netherlands, approval no. EMC 3246 (114‐14‐01).

## AUTHOR CONTRIBUTIONS

L. Utomo performed the animal experiments, processed samples, analyzed and interpreted the data, and wrote the manuscript. N. Fahy processed samples, analyzed and interpreted the data, and wrote the manuscript. N. Kops and S.T. van Tiel supported in sample processing. P.J.M. Leenen supported in data analysis and interpretation. J.H. Waarsing supported in statistical analysis. J.A.N. Verhaar supported the interpretation of data. G.J.V.M. van Osch designed the study, guided the animal experiments, interpreted the data, and edited the manuscript. Y.M. Bastiaansen Jenniskens conceived and designed the study, interpreted the data, and edited the manuscript. All authors have revised the manuscript for important intellectual content and have approved the submission of the manuscript.

## Supporting information


**Supplementary Figure S1: Examples of Pritzker cartilage damage grading**. Examples of 6 grades of cartilage damage on thionin stained sections of mice knees. Assessment criteria are presented in Supplementary Table S1.

Supplementary Figure S2: Gating strategy for synovial macrophages.

Supplementary Figure S3: Gating strategy for peripheral blood monocyte subsets.

**Supplementary Figure S4: Cartilage damage after sham‐surgery or DMM‐surgery**. Thionin staining of the tibia plateaus of mice various days after surgery. Scale bar: 50 µm.

Supplementary Figure S5: Correlations between the Prizker and OARSI scoring methods for structural cartilage damage (*n*=52 DMM‐operated knees).

Supporting information.

Supporting information.
