# Supplementary figures and images for: Macrophage phenotypes and monocyte subsets after destabilization of the medial meniscus in mice
Source: J Orthop Res. 2020 Dec 29;39(10):2270–80. doi: 10.1002/jor.24958 (PMC8518591; doi:10.1002/jor.24958)

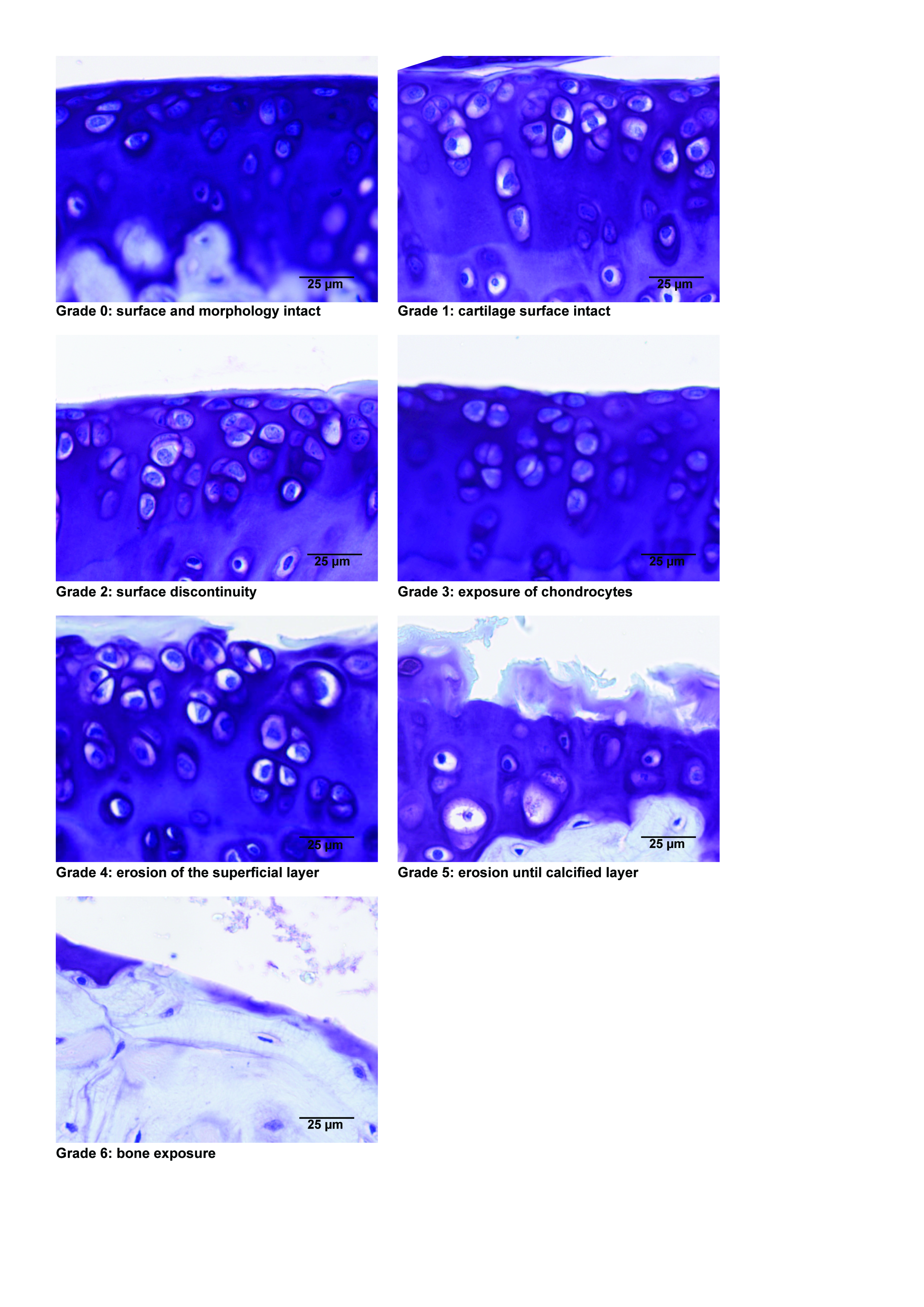

Supplement: Supplementary file 1 — Supplementary Figure S1: Examples of Pritzker cartilage damage grading. Examples of 6 grades of cartilage damage on thionin stained sections of mice knees. Assessment criteria are presented in Supplementary Table S1. [file JOR-39-2270-s006.tif]

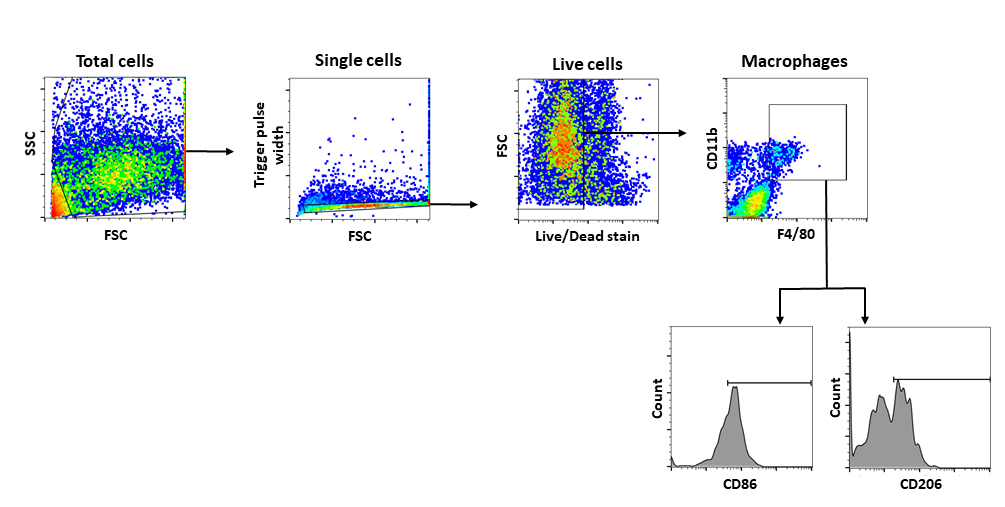

Supplement: Supplementary file 2 — Supplementary Figure S2: Gating strategy for synovial macrophages. [file JOR-39-2270-s005.tif]

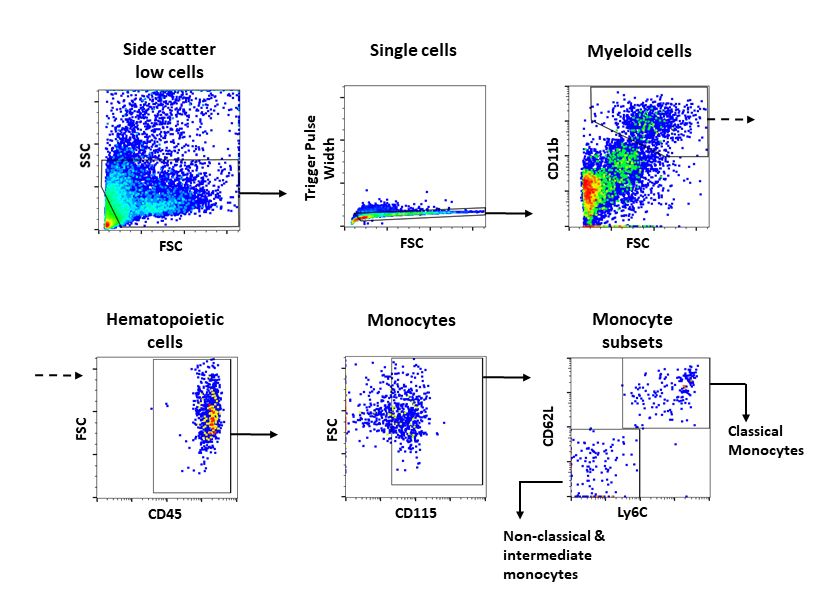

Supplement: Supplementary file 3 — Supplementary Figure S3: Gating strategy for peripheral blood monocyte subsets. [file JOR-39-2270-s001.tif]

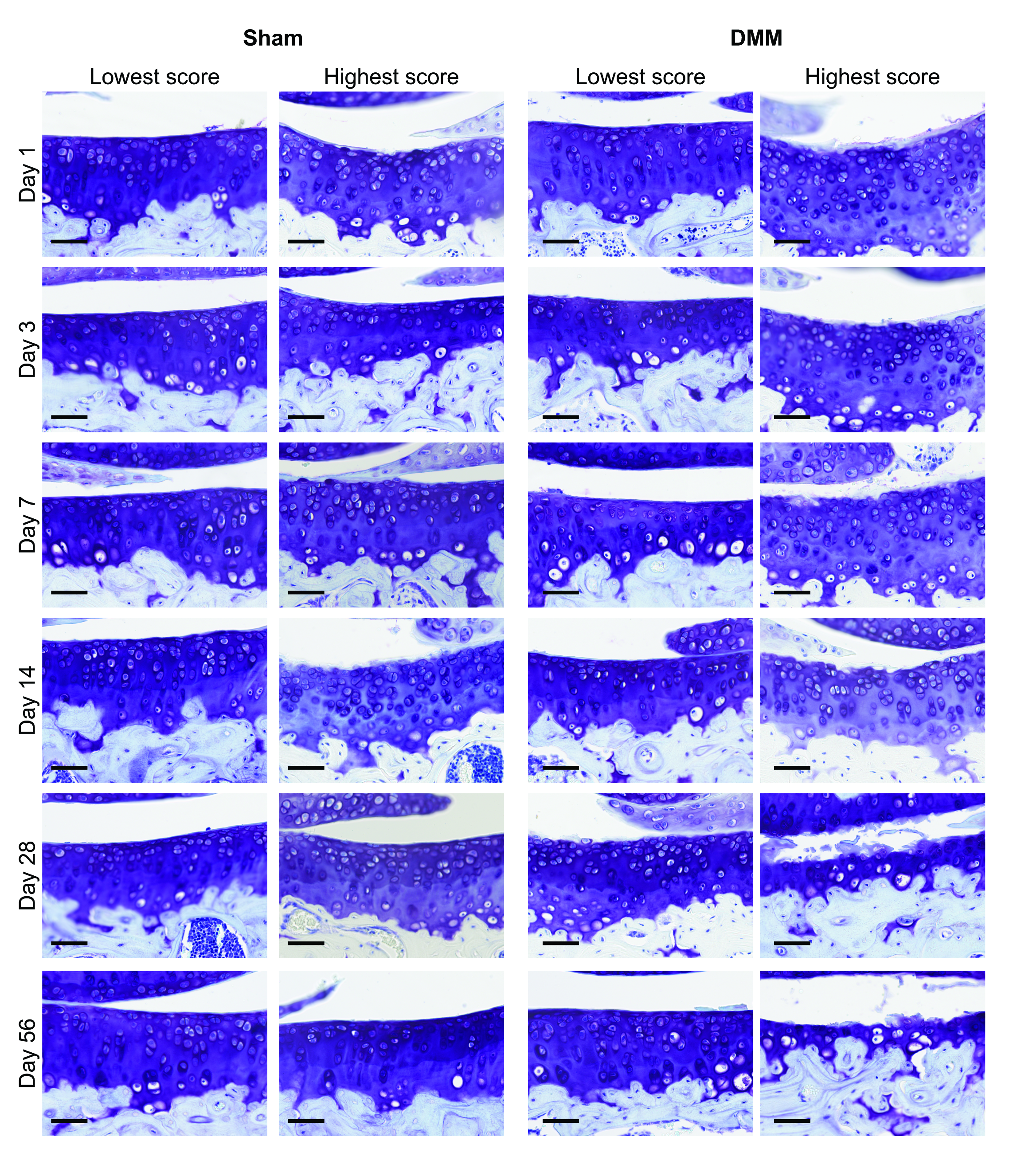

Supplement: Supplementary file 4 — Supplementary Figure S4: Cartilage damage after sham‐surgery or DMM‐surgery. Thionin staining of the tibia plateaus of mice various days after surgery. Scale bar: 50 µm. [file JOR-39-2270-s003.tif]

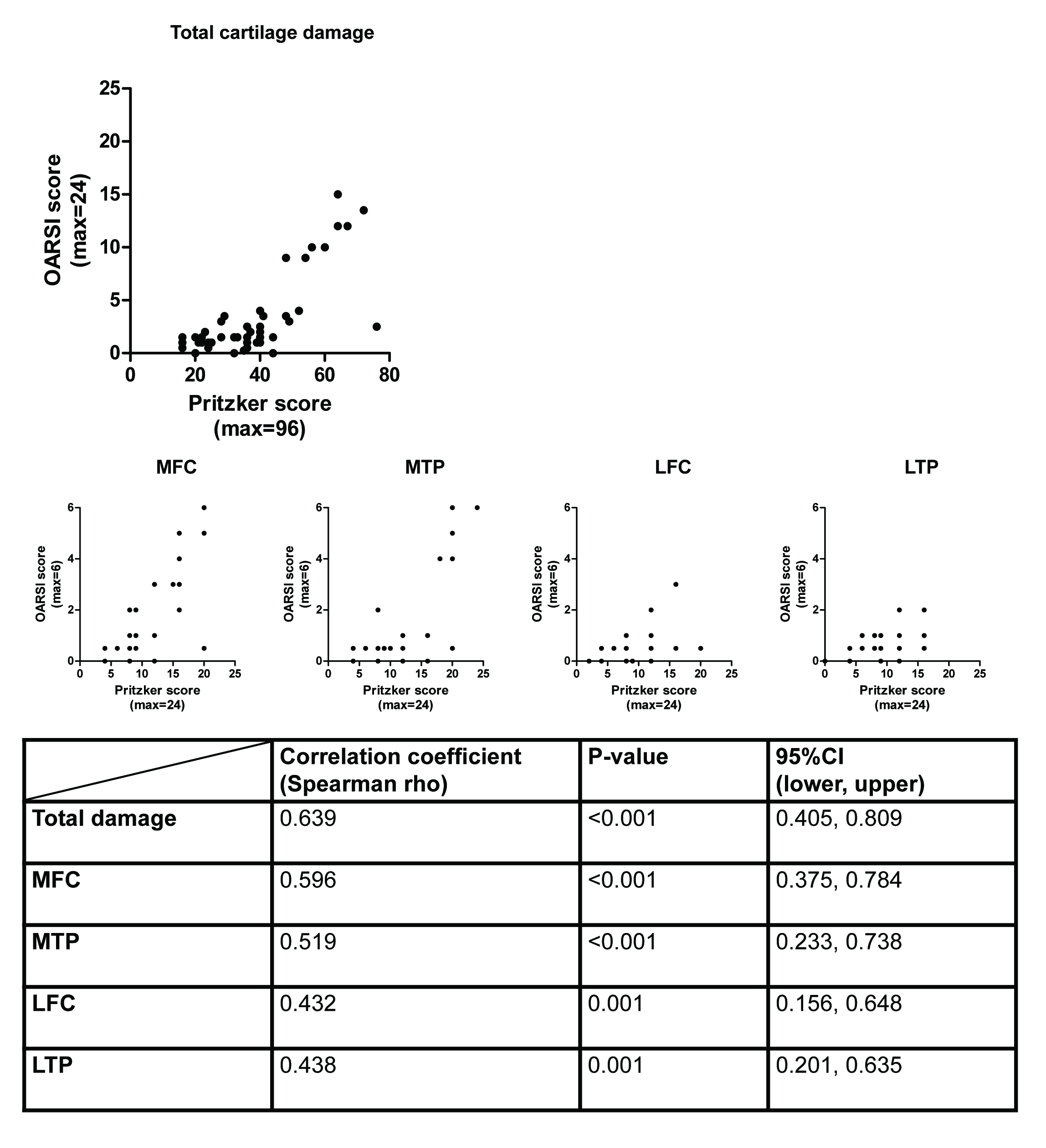

Supplement: Supplementary file 5 — Supplementary Figure S5: Correlations between the Prizker and OARSI scoring methods for structural cartilage damage (n=52 DMM‐operated knees). [file JOR-39-2270-s002.tif]
