# Supplementary material for: Macrophage phenotypes and monocyte subsets after destabilization of the medial meniscus in mice
Source: J Orthop Res. 2020 Dec 29;39(10):2270–80. doi: 10.1002/jor.24958 (PMC8518591; doi:10.1002/jor.24958)
Supplement: Supplementary file 6 — Supporting information. [file JOR-39-2270-s004.docx]

**Supplementary Table S1: The Pritzker and OARSI score for evaluation of structural cartilage damage.** For the Pritzker score, the grade describes the depth progression of cartilage degeneration and the stage describes the extent of the cartilage surface that is affected. The total cartilage damage score = grade x stage.

| **Pritzker score (grade and stage)** | | **OARSI score** | |
| --- | --- | --- | --- |
| **Grade:** | **Assessment:** | **Grade** | **Assessment** |
| 0 | Cartilage surface and morphology intact: cells are intact, superficial layer contains flattened chondrocytes in an aligned orientation | 0 | Normal |
| 1 | Cartilage surface is intact: fibrillation and edema of the superficial layer. Cells are clustering, apoptotic and hypertrophic. | 0.5 | Loss of staining without structural changes |
| 2 | Discontinuity of the cartilage surface: damage to the superficial layer with discontinuity or fissures. Cells are clustering, apoptotic, hypertrophic and chondron columns are disoriented. | 1 | Small fibrillations without loss of cartilage |
| 3 | Exposure of chondrocytes: damage to the superficial layer resulting in exposure of chondrocytes. Cells are clustering, apoptotic, hypertrophic and chondron columns are disoriented.  ± reduced staining intensity coupled with clusters of empty lacunae | 2 | Vertical clefts down the layer immediately below the superficial layer and some loss of surface lamina. |
| 4 | Erosion of the superficial layer: delamination of the superficial layer; loss of the superficial layer of the aligned chondrocytes. Cells are clustering, apoptotic, hypertrophic and chondron columns are disoriented. | 3 | Vertical clefs/erosion to the calcified cartilage extending to <25% of the articular surface |
| 5 | Erosion until the calcified layer: complete delamination of the superficial layer until the calcified layer with exposure to the deep zone. Cells in the deep zone are hypertrophic and apoptotic. | 4 | Vertical clefts/erosion to the calcified cartilage extending to 25-50% of the articular surface |
| 6 | Bone exposure: complete loss of the articular cartilage (all zones) resulting in exposure of the subchondral bone. | 5 | Vertical clefts/erosion to the calcified cartilage extending to 50-70% of the articular surface. |
|  |  | 6 | Vertical clefts/erosion to the calcified cartilage extending to >75% of the articular surface. |
| **Stage:** | **Surface area:** |  |  |
| 0 | 0% |  |  |
| 1 | < 10% |  |  |
| 2 | 10-25% |  |  |
| 3 | 25-50% |  |  |
| 4 | > 50% |  |  |
