# Supplementary material for: Macrophage phenotypes and monocyte subsets after destabilization of the medial meniscus in mice
Source: J Orthop Res. 2020 Dec 29;39(10):2270–80. doi: 10.1002/jor.24958 (PMC8518591; doi:10.1002/jor.24958)
Supplement: Supplementary file 7 — Supporting information. [file JOR-39-2270-s007.docx]

**Supplementary Table S2.** Flow cytometry antibodies used for markers of interest.

| **Antibody** | **Clone** | **Fluorophore** |
| --- | --- | --- |
| ***Peripheral blood analysis*** |  |  |
| Anti-mouse CD45 | 30-F11 | FITC |
| Anti-mouse/human CD11b | M1/70 | PE |
| Anti-mouse CD115 | AFS98 | PerCP/Cy5.5 |
| Anti-mouse Ly6C | HK1.4 | APC/Cy7 |
| Anti-mouse CD62L | MEL-14 | APC |
|  |  |  |
| ***Synovial macrophage analysis*** |  |  |
| Anti-mouse/human CD11b | M1/70 | PerCP/Cy5.5 |
| Anti-mouse F4/80 | BM8 | PE |
| Anti-mouse CD86 | GL-1 | PE/Cy7 |
| Anti-mouse CD206 | C068C2 | APC |
